# Publishing habits and perceptions of open access publishing and public access amongst clinical and research fellows

**DOI:** 10.5195/jmla.2020.751

**Published:** 2020-01-01

**Authors:** Robin O’Hanlon, Jeanine McSweeney, Samuel Stabler

**Affiliations:** Associate Librarian, User Services, Memorial Sloan Kettering Cancer Center Library, Memorial Sloan Kettering Cancer Center, New York, NY, ohanlonr@mskcc.org; Associate Librarian, Scholarly Communications, Memorial Sloan Kettering Cancer Center Library, Memorial Sloan Kettering Cancer Center, New York, NY, mcsweenj@mskcc.org; Hunter College, City University of New York (CUNY), New York, NY, ss5346@hunter.cuny.edu

## Abstract

**Introduction:**

Open access (OA) publishing rates have risen dramatically in the biomedical sciences in the past decade. However, few studies have focused on the publishing activities and attitudes of early career researchers. The aim of this study was to examine current publishing activities of clinical and research fellows and their perceptions of OA publishing and public access.

**Methods:**

This study employed a mixed methods approach. Data on publications authored by Memorial Sloan Kettering Cancer Center fellows between 2013 and 2018 were collected via an in-house author profile system and citation indexes. Journals were categorized according to SHERPA/RoMEO classifications. In-person and telephone interviews were conducted with fifteen fellows to discern their perceptions of OA publishing.

**Results:**

The total percentage of fellows’ publications that were freely available OA was 28.6%, with a relatively flat rate between 2013 and 2018. Publications with fellows as first authors were significantly more likely to be OA. Fellows cited high article processing charges (APCs) and perceived lack of journal quality or prestige as barriers to OA publishing. Fellows generally expressed support for the National Institutes of Health (NIH) public access policy.

**Conclusions:**

While the fellows in this study acknowledged the potential of OA to aid in research dissemination, they also expressed hesitation to publish OA related to confusion surrounding legitimate OA and predatory publications and frustration with APCs. Fellows supported the NIH public access policy and accepted it as part of their research process. Health sciences information professionals could potentially leverage this acceptance of public access to advocate for OA publishing.

## INTRODUCTION

Open access (OA) and public access publishing represent growing and ever-changing areas of interest to health sciences information professionals. The recent ten-year anniversary of the National Institute of Health’s public access policy and the approaching ten-year anniversary of the launch of the National Library of Medicine’s full-text archive PubMed Central (PMC) provide an opportunity to reflect on the growth of public access over the past decade. The emergence of Plan S, an initiative by cOALition S, has stirred debate about the future of OA among publishers, academics, researchers, and information professionals.

While many health sciences information professionals have been at the forefront of open publishing initiatives, the vitality of the OA movement ultimately hinges on the decisions made by authors about where they publish their work. This is particularly applicable in regard to the research and publishing activities of early career professionals (e.g., medical fellows and residents), many of whom are still in the nascent stage of their publishing careers. As early career professionals in the health sciences establish their research careers, it is essential to develop a baseline understanding of how this group perceives the challenges and benefits of various publishing models, not only to understand how these attitudes may shift over time, but also to understand how we as health information professionals can support this group in the immediate future.

Previous studies have sought to examine authors’ perceptions of OA, though most have focused on faculty and none have exclusively examined the experiences and perceptions of early career researchers. Study results have varied with some authors finding age, seniority, and rank of faculty authors were not strong predictors of authors’ perceptions of OA [1], while other studies have found that age did have an impact [2]. Studies have also found that faculty were more likely than graduate students and postdoctoral researchers to believe that OA publications were of lower quality than subscription-based publications [3], yet others have found that older, tenured faculty were more likely to adopt OA practices [4]. The results of studies of the perceptions of OA by discipline have been equally mixed, with some studies stating that attitudes were relatively consistent across the academic community [5] and other studies contending that attitudes varied by discipline [2].

Findings regarding barriers to publishing OA have been more consistent across the literature. Dallmeier-Tiessen et al. analyzed responses from 38,358 authors across multiple disciplines and found that the most common barriers to OA publishing were funding and perceived lack of quality [6]. Respondents in the fields of medicine and the biological sciences were most likely to have paid an OA article processing charge (APC). Other studies have reinforced these findings [7, 8] and have emphasized concerns related to copyright and plagiarism [9] and how OA might affect promotion and tenure [10].

Previous studies have also attempted to determine authors’ perceptions of public access, particularly in regard to the National Institutes of Health (NIH) public access policy [11, 12]. Compared to the study of OA, the literature on researchers’ attitudes toward public access is sparse. There is a substantial gap in the literature in terms of the awareness and perceptions of the NIH public access policy among early career professionals.

Just how much OA publishing is taking place in the sciences has been the subject of many scientific studies. A 2012 study found that that only 6,713 of 340,000 OA scientific articles published in 2011 were immediately OA [13], while a more recent large-scale analysis estimated that at least 28% of the scholarly literature was OA [14]. Biomedicine saw a 16-fold growth of OA publications between 2000 and 2011 [14]. While the OA movement is clearly growing, how much of this growth is due to early career researchers remains unclear.

Early career researchers such as clinical and research fellows are a population in transition, existing at the foundation of their professional careers. This group traditionally conducts and publishes research, but they are still forming their research and publishing praxis and, as such, may provide valuable insight into how publishing habits change over time. The aim of this study was to develop a more nuanced understanding of the publishing activities of early career researchers in the health sciences. Additionally, the study aimed to understand factors that influence fellows’ publication targets, perceived advantages and disadvantages of OA publishing, perceived barriers and abetments to OA publishing, and views of the NIH public access policy.

## METHODS

### Data gathering

A list of all current Memorial Sloan Kettering Cancer Center (MSK) clinical and research fellows (n=296) was supplied by the MSK Human Resources Department to the authors in August 2018. This list included fellows’ names, departments, hire dates, and previous institutions. In November 2018, this study was determined to meet the regulatory exemption for institutional review board (IRB) review by the MSK Human Research Protection Program under 45 CFR 46.101(b).

A database was created in Microsoft Excel to track all publication data, which was later imported to Stata. Publication data for all fellows was first searched using the MSK Library’s in-house author and publication database, Synapse, which contains references for journal articles, books and chapters, conference papers, meeting abstracts, and other items published by authors at MSK. Currently, Synapse includes content from 1994 to present. Synapse is updated monthly using five bibliographic databases: Scopus, Web of Science, BIOSIS Citation Index, CINAHL, and PsycINFO. A team of three information professionals reviews, vets, and uploads citations to Synapse to ensure accuracy and comprehensiveness.

For fellows without Synapse profiles, an author name search was conducted in Scopus and Web of Science. Authors were then matched with their names and previous institutions. If an ORCID identification (ID) was listed in the author’s Scopus profile, the author’s ORCID ID was checked to determine if the author’s current employer was MSK. To ensure accuracy and reduce name ambiguity, fellows were asked via email to confirm (1) if the author profiles found in Scopus and Web of Science that matched their name and previous institution accurately represented their publication record and (2) if all of their publications were accounted for.

Publication types included in this study were published, scholarly, peer-reviewed journal articles. Conference proceedings (i.e., conference abstracts, conference papers), editorials, and book chapters were excluded. The publication years included were 2013 to 2018. Publication information that was captured at the article level included journal names, publication titles, authors, and publication dates. The most recent impact factor for the journal was also captured. Where available, the five-year impact factor was recorded. If the five-year impact factor was not available, the two-year or current year journal impact factor was recorded. Impact factors were located using Journal Citation Reports and Web of Science. Specific authorship information at the article level included if fellows were listed as first author on the publication and if fellows listed MSK or another institution as their affiliation at the time of publication.

All articles were identified by publication model based on the journal they were published in. For the purposes of this study, journals were grouped according to the SHERPA/RoMEO categories. SHERPA/RoMEO is a service provided by the Securing a Hybrid Environment for Research Preservation and Access (SHERPA) organization, which categorizes the copyright and self-archiving policies of journals by color. The SHERPA/RoMEO color categories are [15]:

- Blue: Author can archive post-print (i.e., final draft post-refereeing) or publisher’s version/portable document format (PDF)
- Green: Author can archive pre-print and post-print or publisher’s version/PDF
- Yellow: Author can archive pre-print (i.e., pre-refereeing)
- White: Archiving is not formally supported
- Ungraded/Unavailable: The publisher’s policies have not been checked by SHERPA/RoMEO or the publication was not found in SHERPA/RoMEO

Publication model information for journals was manually identified by consulting the websites of all journals and by checking this information against the SHERPA/RoMEO database.

The ability to access all publications (i.e., freely available or behind a paywall) was then determined. The goal of this step was to determine the current “openness” of a publication. For example, some publications that were published in green journals, which have several options for making a publication “open” (including archiving pre-prints and post-prints and a paid OA option), might still not be available OA. Quality control measures were taken to ensure that publications were legally freely available, rather than available via research sharing platforms such as ResearchGate. First, publications were checked using Unpaywall, a service that harvests “content from legal sources including repositories run by universities, governments, and scholarly societies, as well as open content hosted by publishers themselves” [16]. Unpaywall has been integrated as a browser plugin in Web of Science, and publications were searched first using the Web of Science platform. Next, publications were checked against the “open access” filters in Scopus. As a final step, all publications were manually checked via their journal sites without being authenticated through institutional journal subscriptions.

Lastly, publications were evaluated to determine if they fell under the auspices of the NIH public access policy. Publications were checked in PMC for an NIH Manuscript Submission (NIHMS) system number or a PMCID number and were checked in eRA Commons and NIH RePORTER. The goal of this final step was to determine what percentage of publications had been made openly available due to required compliance with the NIH public access policy and what percentage had been made available OA that did not fall under this funding mandate.

### Interviews

Semi-structured interviews were conducted with fifteen fellows either in person or via telephone between December 2018 and January 2019. Fellows were recruited for interviews via email. Interviews lasted between thirty and forty-five minutes. Incentives were not offered for participation in the interviews. All interviews were recorded, transcribed, and open coded according to emergent themes. Fellows were asked questions regarding factors influencing their publication targets, their perceptions of OA publishing in their specific disciplines, their perceptions of advantages and disadvantages of OA publishing, and factors that have encouraged or inhibited their decisions to pursue OA publishing or their decisions to do so in the future. The supplemental appendix provides a complete list of interview questions. Definitions were provided to authors when necessary.

Follow-up interviews were conducted in July 2019 to enumerate perceptions of public access among the fellows. Twelve of the original fifteen interviewees participated in the follow-up interviews. All twelve of these interviewees had an article that was freely available OA.

## RESULTS

### Publication analysis

Of the 296 clinical and research fellows at MSK, 57 were determined to have had no scholarly, peer-reviewed journal articles published between 2013 and 2018 and were, thus, excluded from the study. The publication information for an additional 21 fellows could not be verified. After these exclusions, the total number of fellows included was 218. The range of total publications for fellows was 1–45. The average number of publications was 6.8. The total number of publications for all fellows was 1,489.

**Figure 1:**
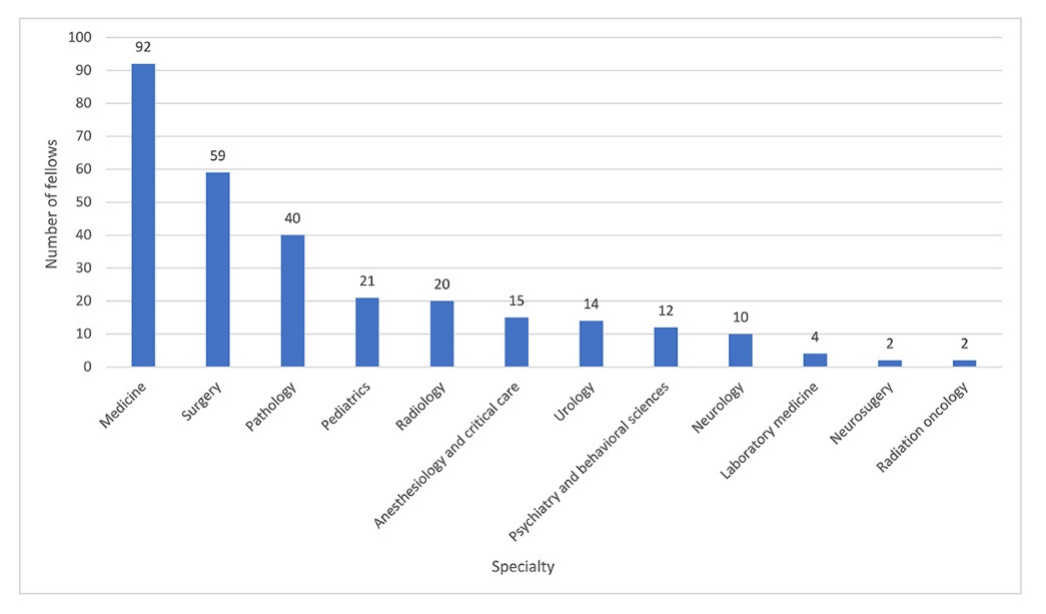
Fellows by discipline

**Figure 2:**
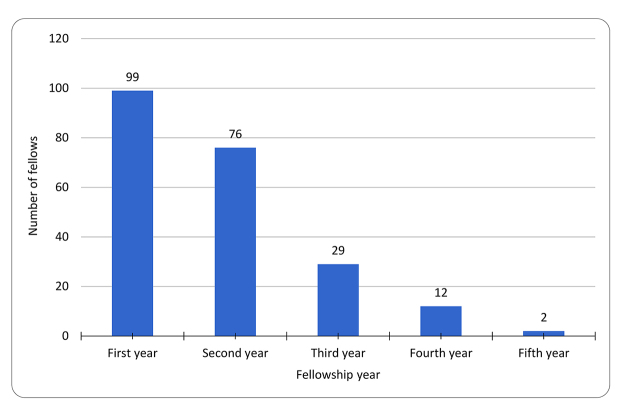
Fellows by research year

The fellows in this study represented 12 clinical and research specialties (Figure 1). Clinical and research specialty information was obtained from the MSK Human Resources Department. The specialty with the highest number of fellows was medicine, followed by surgery and pathology. Most fellows were in their first year of residency, with only 15% of fellows being 3 or more years into their residency programs (Figure 2).

**Figure 3:**
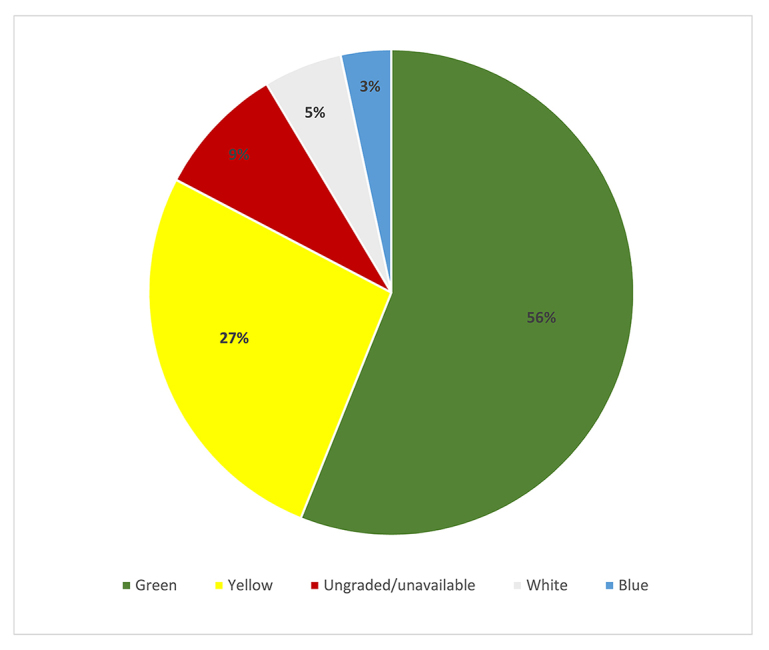
Publications by journal model

The journal publication model with the most publications represented was green, followed by yellow, ungraded/unavailable, white, and blue (Figure 3).

On average, fellows had published 3.8 publications in green journals. Table 1 shows a detailed description of publications by journal type.

**Table 1:** Publications by journal model

Journal model	Range of publications per fellow	Average number of publications per fellow	Total number of publications
Green	0–25	3.8	835
Yellow	0–26	1.8	396
Ungraded/unavailable	0–14	0.5	130
White	0–6	0.4	78
Blue	0–3	0.2	50

Publications included in the analysis appeared in a total of 676 journals. Some (14.9%, n=101) of the journals either did not have an impact factor or the impact factor could not be determined. The impact factors for the remaining journals (n=575) ranged from 0.349 to 79.258. The journal with the highest impact factor was the *New England Journal of Medicine*. Table 2 shows the median impact for each journal category and the top 3 journals for each category by impact factor.

**Table 2:** Journal impact factors

Journal category	Total number	Median impact factor	Top 3 journals by impact factor	

			Journal	Impact factor
Green	389	2.896	*Science*	37.205
			*BMJ Online*	23.562
			*The Lancet Respiratory Medicine*	21.466
Yellow	165	3.483	*Nature*	40.137
			*Nature Medicine*	32.621
			*Cell*	31.398
Ungraded/unavailable	63	1.422	*Journal of the National Comprehensive Cancer Network*	6.471
			*Allergy: European Journal of Allergy and Clinical Immunology*	6.048
			*Biochimica et Biophysica Acta*–*Molecular Cell Research*	3.849
White	34	5.291	*New England Journal of Medicine*	79.258
			*Journal of the American Medical Association (JAMA)*	47.661
			*Nature Reviews Cancer*	37.147
Blue	25	3.746	*American Journal of Respiratory and Clinical Care Medicine*	15.239
			*Haematologica*	9.09
			*Journal of Nuclear Medicine*	6.646

The total percentage of publications that were freely available OA was 28.3% (n=422). The average number of publications per fellow that were freely available OA was 1.9. These data were positively skewed, with 75% of fellows having 3 or fewer publications available OA.

MSK publications (publications where the author or fellow listed MSK as their affiliation) made up 16.2% (n=241) of the total number of publications. The average number of MSK publications that fellows had authored was 1.1. The range of MSK publications for fellows was 0–44.

**Figure 4:**
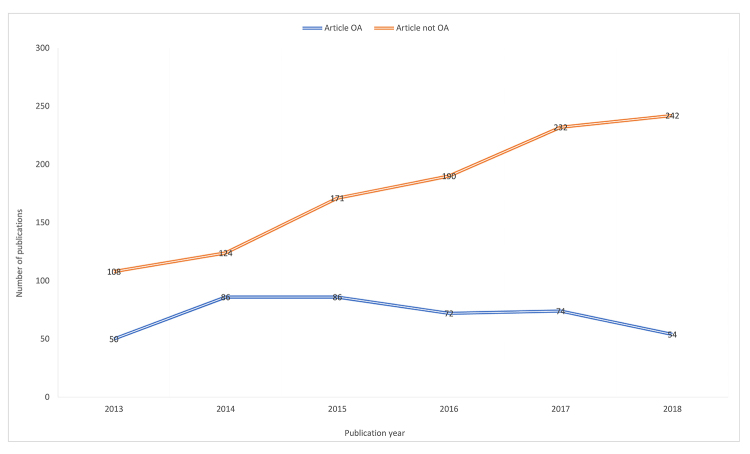
Article availability by publication date

Figure 4 shows the general trend of the availability of publications (available or not available OA) between 2013 and 2018. While the overall number of publications trended upward, the overall number of publications available OA remained relatively flat.

The vast majority of the 422 publications that were freely available (74.9%, n=316) fell under the NIH public access policy, representing 21.2% of all publications in this study. Publications that were freely available without falling under the NIH public access policy accounted for only 7.1% (n=106) of all publications in the study.

A chi-square analysis was performed to determine if there was a statistically significant relationship between high output authors and publication openness. “High output” authors were defined as authors with a number of publications in the 90th percentile, which in this case was 17 publications written by 24 authors. We found that high output authors were less likely than non-high output authors to publish OA (χ^2^(1)=7.2284, *p*=0.007).

We were also interested in determining whether first authorship impacted the availability of publications in terms of OA. Publications on which the fellow was listed as first author accounted for 38.1% of all publications (n=568). We found that publications on which fellows were first author were significantly more likely to be available OA than not available OA (χ^2^(1)=10.9705, *p*=0.001).

Furthermore, we examined the relationship between MSK publications and current publication availability. The MSK Library supports several institutional memberships with OA publishers to offset the cost of APCs, including SpringerOpen and *Proceedings of the National Academy of Sciences of the United States of America (PNAS).* We were curious to determine whether MSK publications were any more likely to be available OA than non-MSK publications. We found that MSK publications were not significantly more likely to be available OA than publications published at other institutions (χ^2^(1)=2.1093, *p*=0.146).

### Interviews

Fifteen fellows representing 7 MSK clinical fellowships were interviewed for this study. The breakdown of authors by department or discipline was: medicine (n=4), surgery (n=4), urology (n=2), pediatrics (n=2), pathology (n=1), psychiatry (n=1), and radiology (n=1). The total number of publications for all interviewed authors was 150, and the average number of publications per author was 10. Of the 15 authors, 11 had a publication that was freely available OA. The average number of OA publications available per interviewed author was 2.

#### Familiarity with open access (OA) and public access concepts

Authors were asked to define “open access” in their own words. Thirteen out of fifteen authors gave adequate descriptions of OA. One author gave an inadequate description, and one author could not provide a definition of OA. The interviewer provided these authors with a definition of OA, from the Scholarly Publishing and Academic Resources Coalition (SPARC), as “the free, immediate, online availability of research articles coupled with the rights to use these articles fully in the digital environment” [17]. All fifteen authors were able to adequately define “article processing charges.”

Twelve of the fifteen authors were asked to define “public access.” Two authors equated “public access” with “open access.” Eight referred to the NIH public access policy, and one asked the interviewer if public access funding was associated with “National Cancer Institute Funding” and, upon further questioning, referred to the NIH public access policy. One author was unable to offer a definition. The NIH public access policy was defined for interviewees as the “policy that ensures that the public has access to the published results of NIH-funded research,” which “requires scientists to submit final peer-reviewed journal manuscripts that arise from NIH funds to the digital archive PubMed Central” [18].

#### Factors influencing publication targets

Authors were asked to describe factors influencing their publication targets. The most frequent factor given for selecting a journal to publish in related to journal readership and was described as “target audience” or “relevance to readers.” Authors noted that they aimed to publish in the journals that were most frequently read in their own disciplines.

Impact factor was also listed as a factor for selecting a journal, with some authors listing specific impact factor values that they would not consider publishing below. One author specifically stated that he would “try to avoid” publishing in a journal with an impact factor below 3.0.

Perceived likelihood of acceptance also influenced journal selection. Authors stated that they were more likely to publish in a journal that they or their coauthors had already published in. Perceived time to publication and duration of the peer-review process were also stated as factors influencing their selections. Authors were more likely to publish in journals that had shorter submission-to-publication windows.

Authors stated that their mentors or the principal investigators of the studies often influenced their target publications, particularly if they were not the first author on the publications. Authors stated that they felt they would be more likely to publish OA if they were first author, consistent with the results of our publication analysis.

#### Overall perceptions of OA publishing

Eleven of the fifteen authors stated that they perceived OA publications as being “of lower quality,” “less prestigious,” and “less credible” than publications that were not OA in their specific disciplines. Authors stated that publications that were fully OA tended to have “lower impact factors.”

Authors questioned whether OA publishing was advantageous in terms of research dissemination due to alternative dissemination channels, citing networking sites like ResearchGate or social media platforms such as Twitter as examples. Authors were unclear of the advantage of initially publishing in an OA publication when funding mandates such as the NIH’s public access policy dictate that authors make their research freely available through PMC twelve months after publication.

Authors distinguished between perceptions of OA in basic science research and clinical research. One author described OA as being viewed “very positively” in basic science disciplines and stated that “pre-print servers like arXiv are rising in popularity with basic science researchers.” Some authors stated that they believed perceptions of various publishing models were more institution-specific than discipline-specific.

#### Overall perceptions of public access publishing

Of the twelve participants who participated in follow-up interviews, ten expressed familiarity with the NIH public access policy. Nine fellows had published articles that were supported by NIH funding, and their associated publications were subsequently deposited in PMC. Of these nine fellows, seven had used submission method C (i.e., depositing a final peer-reviewed manuscript in PMC via NIHMS). Two had used submission method B (i.e., arrangements are made by the publisher to deposit the publication in PMC). No interviewees used submission method A (i.e., publishing in a journal that deposits all final published publications in PMC without author involvement).

Of the nine interviewees who had NIH public access policy publications, all expressed support for the policy. The top reasons for supporting the policy were enhanced research dissemination and a desire to make research more discoverable and equitable. Several authors noted that the policy had been in place throughout their research careers and was essentially “second nature.” One author explained:

I’ve been actively doing research for five years and most of it is NIH funded, so I’m just used to having to adhere to the policy and the paperwork that’s involved. It’s just part of the research process for me.

Two interviewees described challenges with NIH public access policy compliance, including the time-consuming nature of the documentation required but described the policy as “positive overall” and “still important,” respectively.

#### Perceived advantages and disadvantages of OA publishing

Authors listed increased readership as an advantage to publishing OA, stating that OA could aid in “advancing your research agenda” and “research dissemination.” Authors also mentioned the possibility of “increased citation counts” as an advantage. The potential for increased collaboration between institutions was another advantage, with one author stating that “open access can help you more quickly identify people doing similar work.” Finally, authors stated that the “egalitarian” nature of OA was beneficial for both author and reader.

Perceived disadvantages of OA publications included perceived lack of research quality and poor reputations of OA publications among research peers and mentors. Authors also stated that the relative newness of some OA journals was a disadvantage because newer journals tended not to be well established in terms of readership or reputation and tended to have lower impact factors.

#### Perceived barriers and abetments to OA publishing

When asked to identify potential barriers to OA publishing, thirteen of fifteen authors listed APCs. One author stated that he would never pay an APC “on principle.” Other authors expressed willingness to pay an APC but stated that the APCs were simply “too high.” One author summarized this sentiment:

I’d like to publish more in open access journals, but some of the fees for authors are like $3000, $4000. I’m on a trainee salary, living in one of the most expensive cities in the country, I’ve got two kids. How could I ever afford that fee? It makes it untenable. I mean, with my research, I want to be “open,” but with the fees they charge, how can I?

Another author described why she avoided paying author processing charges journals with a paid OA option:

I think all my publications so far have been in a journal where there’s some sort of “author’s choice” where you can pay to have your article be open access, but then you see the cost and the idea that it’s a real “choice” is laughable.

Authors also identified negative associations with OA publishing models as a barrier and noted confusion surrounding predatory publishers and OA publishers. One author stated:

It [OA publishing] really has a negative connotation. In 50% of cases, this connotation is probably inaccurate. We younger researchers are very aware of predatory journals and somewhere along the line these got linked with open access journals.

Another author further elucidated this point:

It’s confusing in terms of which journals are “legitimate” open access and which are “predatory,” if they both require fees.

When asked to identify potential abetments or factors that would further encourage OA publishing, authors listed research funding specifically earmarked for offsetting the cost of APCs. Authors also stated that having more established scientific scholars publish their work OA might encourage early career researchers and trainees to follow suit. One author stated:

There’s a growing movement and the more that prominent scientists publish in them, maybe more and more people will want to publish in them. It’s a virtuous cycle, especially for people of my generation.

Lastly, authors expressed confusion regarding OA model labels, specifically “color” models as presented in SHERPA/RoMEO, and the role of self-archiving in pre-print and post-print repositories in OA.

## DISCUSSION

Many of the perceived barriers and challenges to publishing OA were consistent with those described in previous studies. For instance, Dallmeier-Tiessen et al.’s study found the 2 most commonly cited barriers to OA publishing were funding (39% of respondents) and perceived lack of quality (30% of respondents) [6], which were the 2 most commonly cited barriers in our study. It was worth noting, however, that whereas 39% of the participants in Dallmeier-Tiessen et al.’s study cited funding as a barrier, 86% of fellows in the present study cited funding as a barrier. While the interview sample for this study was small, the publication data of the fellows seemed to align with this cited barrier. Fellows stated that they would not pay an APC “on principle,” and most of the “openly available” published articles in this study were a result of falling under the NIH public access mandate.

The fact that there was a significant relationship between first authorship and publication openness might indicate that early career researchers, if given agency, were more likely to publish OA. However, while 86.0% (n=1,281) of publications in this study were published in blue, yellow, or green journals, only 28.6% of all publications were freely available OA. It was also worth noting that over the course of 5 years, OA rates did not keep pace with publication rates. The fellows in this study published more as they progressed in their careers, but the rate of OA publications remained relatively flat, which might indicate that many authors were not taking advantage of the paid OA option in “hybrid” journals.

Shamash noted that the “average APC continues to rise by £100 a year since 2014” [19]. As an example, during the process of conducting this study, the APC for *American Journal of Transplantation* (a Wiley title) rose from $4,200 to $4,300 USD. APCs that continue to rise in price may create equity issues in scientific scholarly publishing, serving to further widen the gap between the “haves” and “have nots,” in terms of research funding.

High APCs can be particularly problematic for early career researchers and trainees who lack the requisite funding to pay for APCs. Björk’s 2017 study highlighted the staggering transformation of subscription journals to “hybrid OA” journals, from around 2,000 in 2009 to almost 10,000 in 2016 (a 400% increase) [20]. The genuine openness of these “hybrid OA” journals, though, is questionable given that high APCs create barriers to publishing in them, leading authors to look elsewhere or to opt out of the “open” option. The lack of growth in OA publications may also be the result of early career researchers not “opting in” to self-archiving options where available.

Continued education on the part of information professionals may help to offset misconceptions about OA, but ultimately a paradigm shift in the scholarly communications infrastructure may be necessary to help early career professionals in the health sciences not only understand the benefits of OA, but also have the ability to take advantage of OA publishing to its fullest extent. OA advocacy by information professionals can only go so far without the full institutional support of research leaders, administrators, and faculty.

Effective advocacy also requires dismantling pre-existing power structures that promote inequity in the scholarly publishing sphere, which includes high APCs. For years, information professionals have worked to offset the cost of APCs for researchers through OA funding, and many researchers have “built” the cost of APCs into funding. Yet perhaps the time has come for information professionals to present authors with research that illustrates the rapid growth of hybrid APCs and the overall lack of hybrid APC pricing transparency among publishers. In 2016, the Norwegian Research Council and German Research Foundation opted to pay OA fees for researchers but prohibited them from being spent on articles in hybrid journals [21]. Research Libraries UK describes the hybrid model as a form of “double dipping,” wherein “a publisher seeks an unwarrantable increase in revenues by levying article processing charges (APCs) for publication in a hybrid journal, while not providing a proportionate decrease in subscription costs” [22]. Information professionals should stay abreast of developments that highlight the “double dipping” nature of hybrid models and educate their constituents about why this model may be problematic. Early career professionals who lack the funding of senior researchers may be ideal partners for challenging the current “paid OA” model in hybrid publications.

In addition to “growing up” with the public access funding mandates, early career researchers have effectively grown up with predatory publishing. The rise of predatory publishing has only served to exacerbate concerns about the quality of OA publications and has resulted in a general state of wariness and distrust of OA. The fellows have spent their early research years struggling to differentiate legitimate OA publications from predatory publications, leading to confusion surrounding not just what OA is, but why it matters. For many early career researchers, the motivation to publish their research OA is simply not there, especially when so much of their research falls under the domain of public access.

Dawson offers several practical suggestions for effective strategies for OA outreach, such as eliminating OA jargon and collaborating with research units in our institutions [23]. She also suggests that information professionals should attempt to focus their efforts on OA advocacy as much, if not more, than they do on enforcing compliance for publications under specific funding mandates. This argument seems especially salient given that, in this study, some fellows seemed to equate public access with OA or had little interest in openness beyond public access, with one fellow stating:

Most of my research is supported by NIH, so it ends up being “open” no matter what I do. I don’t really care how it happens. It feels good that tax payers get access to research they funded, I approve of that. But I feel no strong desire to make my work that is not affiliated with NIH funding open, especially if it means I have to pay a fee.

Fellows expressed support for the NIH public access policy. Health sciences information professionals could potentially leverage the fact that, today, early career professionals have essentially grown up with a policy that effectively mandates “openness.” These fellows seemed to understand the benefits of public access but were less certain of the benefits of OA. Health sciences information professionals can draw helpful parallels between OA and public access for researchers.

It is equally as important for information professionals to understand the perspective of early career researchers and trainees when it comes to their motivations for selecting their publication targets and specific publishing models. In the sciences and medicine, factors such as research impact metrics and journal prestige or ranking remain paramount for authors. Information professionals must strive to understand how the historical power dynamics in the field of scientific publishing function and how these systems can create structural inequalities for early career researchers, as well as traditionally marginalized groups. Only by truly understanding the challenges that early career researchers face, as well as their motivations, can we begin to shift the tide toward more open and accessible research outputs.

## SUPPLEMENTAL FILE

Appendix Interview guide

## 

**Robin O’Hanlon**, ohanlonr@mskcc.org, http://orcid.org/0000-0003-0869-4118, Associate Librarian, User Services, Memorial Sloan Kettering Cancer Center Library, Memorial Sloan Kettering Cancer Center, New York, NY

**Jeanine McSweeney**, mcsweenj@mskcc.org, Associate Librarian, Scholarly Communications, Memorial Sloan Kettering Cancer Center Library, Memorial Sloan Kettering Cancer Center, New York, NY

**Samuel Stabler**, ss5346@hunter.cuny.edu, Hunter College, City University of New York (CUNY), New York, NY
