# Supplementary material for: Publishing habits and perceptions of open access publishing and public access amongst clinical and research fellows
Source: J Med Libr Assoc. 2020 Jan 1;108(1):47–58. doi: 10.5195/jmla.2020.751 (PMC6919981; doi:10.5195/jmla.2020.751)
Supplement: Appendix [file jmla-108-47-s001.pdf]

Journal of the Medical Library Association

Supplemental content to
J Med Libr Assoc. Jan;108(1):dx.doi.org/10.5195/jmla.2020.751
www.jmla.mlanet.org
© O'Hanlon, McSweeney, Stabler 2020

Publishing habits and perceptions of open access publishing and public access amongst clinical and research fellows

Robin O'Hanlon; Jeanine McSweeney; Samuel Stabler

APPENDIX

Interview guide

1. What is your research area?

2. How many years have you been conducting research?

3. What are the most important factors to you when choosing a publication target?

4. a) Please define "open access publishing" in your own words.

b) Please define "author processing charges" in your own words.

5. a) Have you published in an open access journal?

b) If yes, how did you hear about this journal and what influenced your decision to publish in an open access journal?

6. What do you feel are advantages and disadvantages of publishing research in open access publications?

7. Describe factors that have encouraged or inhibited your decision to publish in open access publications in the past or that you feel may encourage or inhibit you in the future.

8. Have you used other authors' scientific works that are freely available on the web? If so, describe your usage.

9. In the past, have much have your coauthors, peers, mentors, or principal investigators influenced your choice of journal?

10. How do you feel open access publishing is perceived in your research area?
